# Quantitative T_1_, T_2_, and T_2_* Mapping and Semi-Quantitative Neuromelanin-Sensitive Magnetic Resonance Imaging of the Human Midbrain

**DOI:** 10.1371/journal.pone.0165160

**Published:** 2016-10-21

**Authors:** Takashi Hashido, Shigeyoshi Saito

**Affiliations:** 1 Division of Radiology, Department of Medical Technology, Osaka University Hospital, Suita, Osaka, Japan; 2 Department of Medical Engineering, Division of Health Sciences, Osaka University, Graduate School of Medicine, Suita, Osaka, Japan; Shenzhen institutes of advanced technology, CHINA

## Abstract

**Purpose:**

Neuromelanin is a dark pigment granule present within certain catecholamine neurons of the human brain. Here, we aimed to clarify the relationship between contrast of neuromelanin-sensitive magnetic resonance imaging (MRI) and MR relaxation times using T_1_, T_2_, and T_2_* mapping of the lower midbrain.

**Methods:**

The subjects were 14 healthy volunteers (11 men and 3 women, mean age 29.9 ± 6.9 years). Neuromelanin-sensitive MRI was acquired using an optimized T_1_-weighted two-dimensional (2D)-turbo spin-echo sequence. To quantitatively evaluate the relaxation time, 2D-image data for the T_1_, T_2_, and T_2_* maps were also acquired. The regions of interest (substantia nigra pars compacta [SNc], superior cerebellar peduncles [SCP], cerebral peduncles [CP], and midbrain tegmentum [MT]) were manually drawn on neuromelanin-sensitive MRI to measure the contrast ratio (CR) and on relaxation maps to measure the relaxation times.

**Results:**

The CR in the SNc was significantly higher than the CRs in the SCP and CP. Compared to the SCP and CP, the SNc had significantly higher T_1_ relaxation times. Moreover, the SNc had significantly lower T_2_ and T_2_* relaxation times than the other three regions (SCP, CP, and MT). Correlation analyses showed no significant correlations between the CRs in the SNc, SCP, and CP and each relaxation time.

**Conclusions:**

We demonstrated the relationship between the CR of neuromelanin-sensitive MRI and the relaxation times of quantitative maps of the human midbrain.

## Introduction

Neuromelanin, the dark pigmented granules present in the human central nervous system, was discovered in the 1930s [1]. Neuromelanin is present within certain catecholamine neurons of the human brain, such as the dopamine and noradrenaline-containing neurons of the substantia nigra pars compacta (SNc) [1, 2]. Until recently, it was thought that neuromelanin did not serve any function. However, it is now believed that it plays a vital role in preventing cell death in the brain. Neuromelanin has often been a focus of Parkinson’s disease (PD) research. PD is a neurodegenerative disorder caused by the selective death of pigmented SNc neurons [3, 4], which leads to dopamine depletion in the neostriatum [5] and results in a clinical syndrome involving tremor, rigidity, and impaired motility. In 1988, it was reported that the neuromelanin-containing cells of the SNc are more vulnerable in PD [6]. Therefore, neuromelanin of the human midbrain could be a marker of neurodegenerative diseases.

Neuromelanin has the ability to combine with metals, such as iron, and become a paramagnetic agent [2, 7]. Magnetic resonance imaging (MRI) experiments using T_1_-weighted image (T_1_WI) have revealed that the melanin pigment has T_1_-shortening effects due to bound paramagnetic metals [7, 8]. A previous report described that the death of neuromelanin-containing neurons in the SNc and locus coeruleus is linked to PD and showed that these cells can be visualized *in vivo* with neuromelanin-sensitive T_1_WI [9]. Recently, some neuromelanin-sensitive MRI sequence methods have been proposed for diagnosing neurodegenerative diseases [8, 10, 11], with magnetic transfer contrast (MTC) or T_2_ contrast MRI being better at delineating the SNc [12]. However, these methods are not quantitative, and they apply a semi-automated quantitative analysis for estimating the volumes and MR signal contrast-to-noise ratio. Therefore, the MR contrasts for neuromelanin-sensitive MRI are still being discussed [12].

Mapping the T_1_, T_2_, and T_2_* relaxation times enables quantitative comparison of MRI across multiple subjects and scanners. The fast measurement of the spin-lattice relaxation time constant (T_1_) has been popular for determining pathology in brain tissues [13, 14]. For example, the Look-Locker (LL) imaging sequence is commonly used for rapid T_1_ measurement because it can acquire data quickly [15–18]. In addition, spin-spin relaxation time constant (T_2_) measurements were made with a standard spin-echo (SE) sequence [15]. *Ex vivo* MRI was used to investigate alterations in T_2_ related to Alzheimer’s disease (AD) pathology and other types of neuropathology common in old age [19, 20]. Furthermore, T_2_* relaxation refers to the decay of transverse magnetization caused by a combination of spin-spin relaxation and magnetic field inhomogeneity. T_2_* values are always shorter than the underlying T_2_ values. T_2_* relaxation is seen with gradient-echo imaging because the transverse relaxation caused by magnetic field inhomogeneities is eliminated by the 180° pulse during SE imaging [21, 22]. These quantitative relaxation maps isolate the contributions of individual MR contrast mechanisms (T_1_, T_2_, and T_2_*). Because they provide an unbiased metric for comparing MR scans, quantitative relaxation maps have been used to reveal the relationship between MR maps and histological evaluations in some disease models [17, 18].

The aim of this study was to clarify the relationship between the neuromelanin contrast in neuromelanin-sensitive MRI and the quantitative relaxation times using T_1_, T_2_, and T_2_* mapping of the healthy human midbrain. We hypothesized that this contrast would reflect the T_1_ or T_2_ relaxation times measured by quantitative mapping.

## Materials and Methods

### Subjects

This study was performed in accordance with the World Medical Association’s Declaration of Helsinki and approved by the local institutional review board (Approval No. 14195, Osaka University Ethics Committee). Written informed consent was obtained by all subjects. The subjects in this study were 14 healthy volunteers (11 men and 3 women, mean age 29.9 ± 6.9 years). These volunteers had never been diagnosed with PD, schizophrenia, or other similar conditions in the past. Any information about the volunteers’ identities was removed from the images and this information was not used in the analysis or interpretation of the data.

### Imaging protocols

All MRI data were obtained using a 3-Tesla MRI scanner (Achieva, Philips Medical Systems, Best, Netherlands) with a 32-channel head coil, in the form of oblique axial images that were acquired perpendicular to the brainstem. A three-dimensional (3D)-turbo field-echo (TFE) sequence for neuromelanin-sensitive T_1_WI was acquired as reference images. These images were not used for data analysis. To visualize and identify the bilateral SNc, which is called the neuromelanin-sensitive T_1_ contrast of the midbrain, the slice having the largest bilateral SNc areas (hyperintense areas) on the 3D-TFE T_1_WI was determined and set as the center of five consecutive slices on an optimized, T_1_WI, two-dimensional (2D)-turbo spin-echo (TSE) sequence without MTC preparation pulse. This sequence is able to obtain a TSE-related MTC because it provides the equivalent magnetization transfer effect by refocusing pulse [23–25]. The imaging parameters were as follows: repetition time (TR)/echo time (TE) = 623/13 ms, flip angle (FA) = 90 degrees, field of view (FOV) = 180 mm, slice thickness/gap = 2.0/0.2 mm. The central slice of the five consecutive slices on the 2D-neuromelanin-sensitive MRI was used for the measurements. In order to quantitatively evaluate the relaxation time, single slices were also acquired for the T_1_, T_2_, and T_2_* maps. The location of these slices was the same as the location of the central slice on the 2D-neuromelanin-sensitive MRI. The MRI data sets for the T_1_, T_2_, and T_2_* maps were acquired using an LL sequence [26] based on an inversion recovery turbo field-echo (IR-TFE) sequence, a TSE sequence, and a TFE sequence, respectively. Moreover, to reduce any head motion during MRI acquisition, positioning pads were placed at the left and right side of the head in all subjects. All MRI data were collected with different sequences. The main imaging parameters of each sequence are described in Table 1.

**Table 1:** Scan parameters used to acquire the 2D-neuromelanin-sensitive MRI and each quantitative map (T_1_, T_2_, and T_2_*).

	Neuromelanin-sensitive MRI	Maps		
		T_1_	T_2_	T_2_*
Scan sequence (Fast imaging mode)	TSE	IR-TFE	TSE	TFE
TSE or TFE factor	5	15	15	-
Echoes	1	1	5	32
TR [ms]	623	11	3000	640
TE [ms]	13	5.6	20 ^#2^	2.3 ^#3^
TI delay [ms]	-	86.1	-	-
Trigger delay max./act. [ms]	-	700/97.8 ^#1^	-	-
FA [deg]	90	7	90	28
FOV [mm]	180	180	180	180
Slices	5	1	1	1
Slice thickness/gap [mm]	2.0/0.2	2.0/0.0	2.0/0.0	2.0/0.0
Reconstruction voxel matrix per slice [mm]	0.18/0.18/2.00	0.35/0.35/2.00	0.35/0.35/2.00	0.35/0.35/2.00
NSA	10	4	1	2
Scan duration [mm:ss]	07:18	06:48^#4^	03:42	02:45

MRI, magnetic resonance imaging; TSE, turbo spin-echo; IR-TFE, inversion recovery turbo field-echo; TFE, turbo field-echo; TR, repetition time; TE, echo time; TI, inversion time; FA, flip angle; FOV, field of view; NSA, number of signals averaged; ECG, electrocardiogram; RR interval, R wave to R wave interval.

^#1^ from 98 ms, phase interval 172.1 ms, 24 phases;

^#2^ from 20 to 140 ms, step 30 ms, 5 echoes;

^#3^ from 2.3 to 74 ms, step 2.3 ms, 32 echoes.

^#4^ dummy ECG pulse: on,

Number of RR intervals: 1, Entered heart rate: 60 bpm.

### Data processing

We obtained oblique axial images perpendicular to the brainstem and used the slice at the level of lower midbrain for region of interest (ROI) measurements. For semi-quantitative evaluation of the 2D-neuromelanin-sensitive MRI, the ROIs for measuring the signal intensity (SI) were traced manually around the high-signal areas of the bilateral SNc on a single slice, as the SNc neuromelanin-sensitive region. In addition, we measured the SIs in the other regions (bilateral superior cerebellar peduncles [SCP], bilateral cerebral peduncles [CP], and midbrain tegmentum [MT]) of the lower midbrain on the same slice so as to better interpret the characteristics of neuromelanin-sensitive MRI [27]. These ROI measurements were performed using the ImageJ software (1.48v, National Institutes of Health, MD, USA). The semi-quantitative contrast ratios (CRs) of three regions (SNc, SCP, and CP) were calculated relative to the average SI values in the MT [28], as follows:

$$CR_{i}=\left(SI_{i}\;-\;SI_{MT}\right)/SI_{MT},\text{ where }i=SNc,\;SCP,\;and\;CP$$

**Fig 1:**
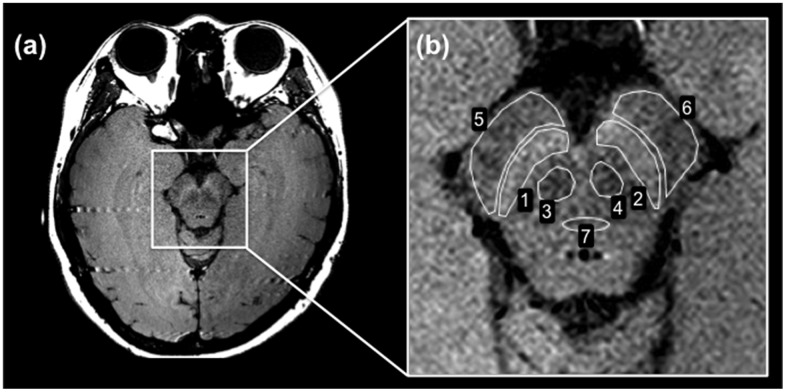
Neuromelanin-sensitive MRI of the lower midbrain. (a) 2D-neuromelanin-sensitive MRI. The SNc appears as the high SI area. The SCP is the small low SI area below the SNc. The CP is above the SNc. The MT is located in the middle of the lower midbrain. (b) The ROIs for measuring the SI were traced manually at the following locations on a single slice: SNc (ROIs 1 and 2), SCP (ROIs 3 and 4), CP (ROIs 5 and 6), and MT (ROI 7).

For measuring the T_1_, T_2_, and T_2_* relaxation times, each map was generated using the Relaxation Maps Tool (Ver. 2.1.1, Philips Medical Systems, Best, Netherlands) supported by the PRIDE environment (Philips Medical Systems). Since the software used to analyze the neuromelanin-sensitive MRI (ImageJ) and T_1_, T_2_, and T_2_* maps (Relaxation Maps Tool) was not the same, we traced the ROIs on the relaxation maps in a similar way to the neuromelanin-sensitive MRI. However, the SNc was difficult to distinguish from the substantia nigra pars reticulata (SNr) on the relaxation maps. A previous report by Menke et al. parceled the substantia nigra into the SNr and SNc by using connectivity information from diffusion tensor imaging [29]. A different report by Langley et al. delineated and segmented the substantia nigra on susceptibility weighted and neuromelanin-sensitive images by using structural information [30]. The later concluded that the delineated hyperintense substantia nigra area on the neuromelanin-sensitive image occupies similar spatial locations and orientations as the SNc defined in Menke et al. Therefore, in order to take measurements corresponding only to the SNc, we used the defined ROIs of the SNc on the neuromelanin-sensitive MRI as a reference (Fig 1b), so as to manually trace the same ROIs (location, shape, and size) on the relaxation maps. The ROIs of the SCP, CP, and MT were also traced in a similar way to the neuromelanin-sensitive MRI (Fig 1b). In order to correctly trace the ROIs, the gray scale was changed to color-contrast scale, when it was necessary, so as to have a better visualization. The ROIs were basically defined on the neuromelanin-sensitive MRI, and then they were applied on the relaxation maps. Each relaxation map had same ROIs for the SNc, SCP, CP, and MT, respectively. Furthermore, before drawing the ROIs, the background noise in each map was reduced using the manual ROI setting on this software. If image noise reduction was difficult with the manual ROI setting, we used the automatic noise reduction. All ROI measurements were performed by a single author (TH) and were confirmed by the other one (SS) in order to reduce user bias.

### Statistical analysis

All of the values in all of the groups are expressed as the mean ± the standard deviation. To determine the differences of the CRs among three regions (SNc, SCP, and CP) and relaxation times among all regions (SNc, SCP, CP, and MT), Kruskal-Wallis tests and multiple comparisons as a post-hoc test (Dunns method) were performed using Prism 5 (Version 5, GraphPad Software, CA, USA). Linear regression analyses were used to assess the correlations between each relaxation time and the CRs in the SNc, SCP, and CP, respectively. For these analyses, the average values of the relaxation times and the CRs in the left and right side of each region were used. We searched for significant correlations between the T_1_, T_2_, and T_2_* values and the CRs in each region using Pearson product-moment correlation analyses. A p value of <0.05 was considered significant.

## Results

**Fig 2:**
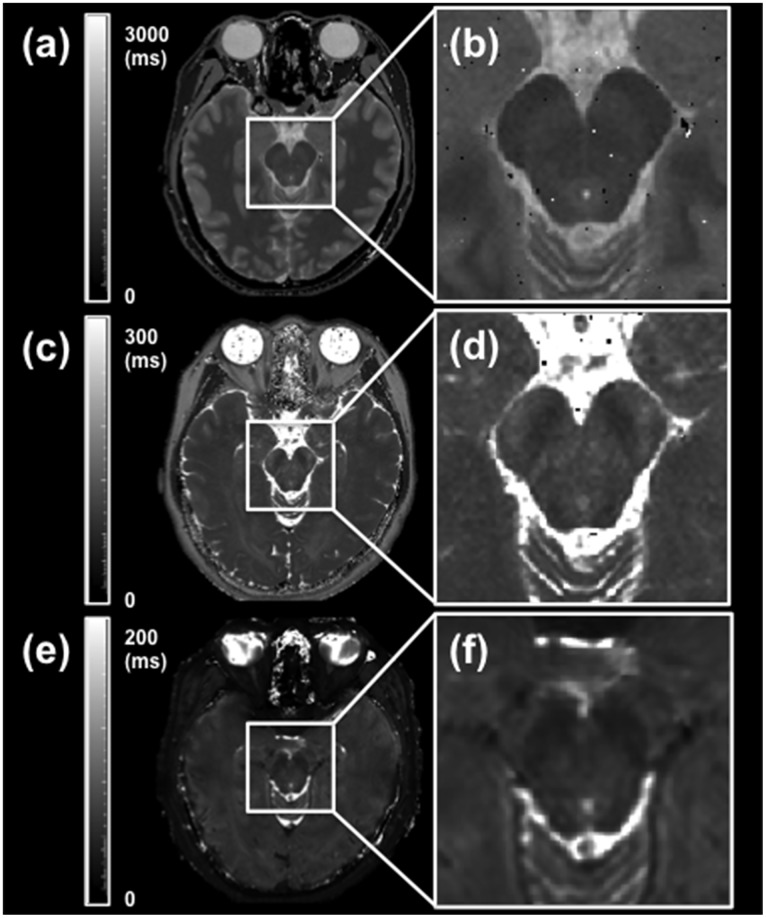
Typical quantitative T_1_, T_2_, and T_2_* maps of the lower midbrain. (a) Typical T_1_ map. The gray scale represents the T_1_ values from 0 to 3000 ms. (b) Enlarged T_1_ map. (c) Typical T_2_ map. The gray scale represents the T_2_ values from 0 to 300 ms. (d) Enlarged T_2_ map. (e) Typical T_2_* map. The gray scale represents the T_2_* values from 0 to 200 ms. (f) Enlarged T_2_* map. The location of the single slices of the T_1_, T_2_, and T_2_* maps was the same as the location of the neuromelanin-sensitive MRI slice.

Fig 1 shows a typical 2D-neuromelanin-sensitive MRI of the lower midbrain of healthy volunteers (Fig 1a), and a typical ROI measurement for the semi-quantitative evaluation of the CRs (Fig 1b). The seven ROIs used for measuring the SI were drawn at seven locations on a single slice (Fig 1b). The ROI numbers represent the SNc (ROIs 1 and 2), SCP (ROIs 3 and 4), CP (ROIs 5 and 6), and MT (ROI 7). Neuromelanin-sensitive MRI of the lower midbrain showed high SI areas that corresponded to the bilateral SNc (Fig 1b, ROIs 1 and 2). The bilateral SCP regions and CP regions were distinguishable as lower SI areas compared with the bilateral SNc regions (Fig 1b, ROIs 3–6). Typical quantitative T_1_, T_2_, and T_2_* maps for healthy volunteers are shown in Fig 2a, 2c and 2e. Quantitative maps for all 14 subjects were available for analyses in this study. These enlarged maps are shown in Fig 2b, 2d and 2f.

**Fig 3:**
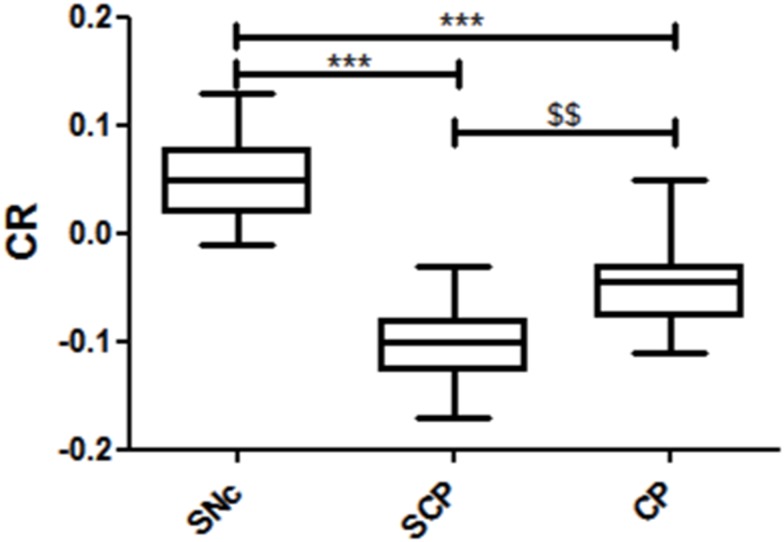
Measurements of CR of the lower midbrain. The box plot represents the minimum, first quartile, median, third quartile, and maximum of the CR. *** p < 0.001, vs. the CR in the SNc; $ $ p < 0.01, vs. the CR in the SCP.

In the semi-quantitative analysis, the CRs in the three regions (SNc, SCP, and CP) were calculated by using the SIs of neuromelanin-sensitive MRI. The CR averages in the SNc, SCP, and CP were 0.056 ± 0.039, -0.100 ± 0.032, and -0.049 ± 0.038, respectively. The CR in the SNc had a higher signal compared to the other two regions (Fig 3, p < 0.001). Additionally, the CR in the SCP had a lower signal compared to the CP (Fig 3, p < 0.01). The CR in the SNc was higher than the CR in the CP, which in turn was higher than the CR in the SCP.

**Fig 4:**
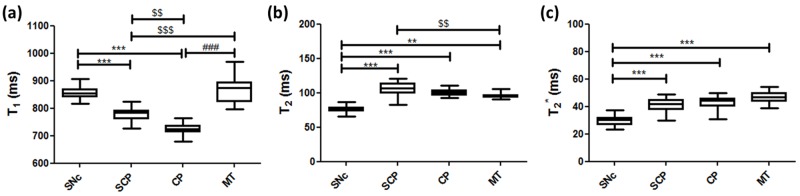
Measurements of quantitative T_1_, T_2_, and T_2_* relaxation times of the lower midbrain. (a) T_1_ relaxation times. (b) T_2_ relaxation times. (c) T_2_* relaxation times. The box plot represents the minimum, first quartile, median, third quartile, and maximum of each relaxation time in the four regions. ** p < 0.01 and *** p < 0.001, vs. the relaxation times in the SNc; $ $ p < 0.01 and $ $ $ p < 0.001, vs. the relaxation times in the SCP; ### p < 0.001, vs. the relaxation times in the MT.

Fig 4 shows the measurements of each quantitative T_1_, T_2_, and T_2_* relaxation time of the lower midbrain. The T_1_ values in the SNc, SCP, CP, and MT were 856.4 ± 24.3 ms, 777.8 ± 23.9 ms, 724.8 ± 19.6 ms, and 872.5 ± 47.6 ms, respectively. The T_1_ relaxation times in the SNc were higher than the relaxation times in the SCP and CP regions (Fig 4a, p < 0.001). In addition, the T_1_ relaxation times in the SCP were higher than the relaxation times in the CP (Fig 4a, p < 0.01), and lower than those in the MT region (Fig 4a, p < 0.001). The T_1_ relaxation times in the CP were also lower than the relaxation times in the MT region (Fig 4a, p < 0.001). The T_2_ values in the SNc, SCP, CP, and MT were 76.1 ± 4.9 ms, 106.5 ± 9.0 ms, 100.4 ± 4.4 ms, and 95.9 ± 4.1 ms, respectively. The T_2_ relaxation times in the SNc were lower than the relaxation times in the SCP, CP (Fig 4b, p < 0.001), and MT regions (Fig 4b, p < 0.01). The T_2_ relaxation times in the SCP were higher than the relaxation times in the MT region (Fig 4b, p< 0.01). The T_2_* values in the SNc, SCP, CP, and MT were 30.0 ± 3.5 ms, 41.3 ± 5.0 ms, 42.3 ± 5.6 ms, and 46.9 ± 4.2 ms, respectively. The T_2_* relaxation times in the SNc were lower than the relaxation times in the other three regions (Fig 4c, p < 0.001). Furthermore, all regions except the SNc had similar T_2_* values with no significant differences (Fig 4c). The T_2_* relaxation times had the same tendency as the T_2_ relaxation times (Fig 4b and 4c).

**Fig 5:**
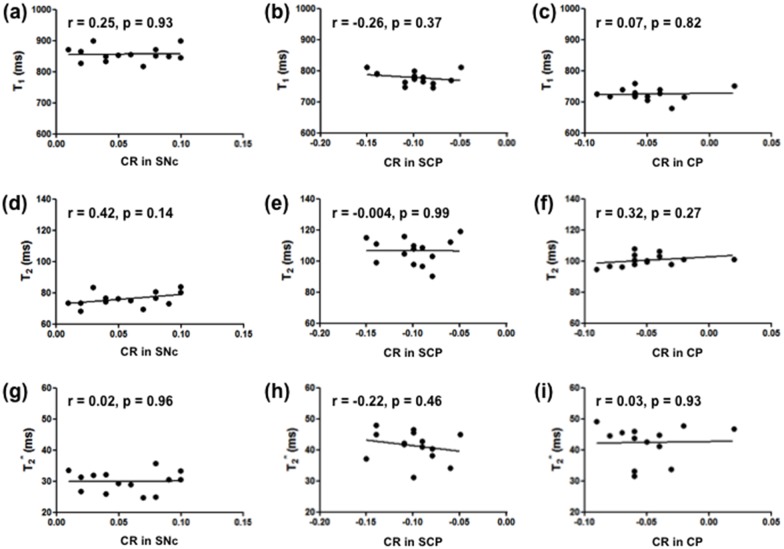
Correlations between the CR and quantitative T_1_, T_2_, and T_2_* relaxation times. (a, d, g) Correlation between each relaxation time and the CR in the SNc. (b, e, h) Correlation between each relaxation time and the CR in the SCP. (c, f, i) Correlation between each relaxation time and the CR in the CP. The average values of the CRs and relaxation times in the left and right side of each region were used.

Fig 5 shows the results of the correlation analyses between the quantitative relaxation times and the semi-quantitative CRs in the SNc, SCP, and CP of the lower midbrain. There were no significant correlations between the CRs in the SNc, SCP, and CP and each relaxation time.

## Discussion

This study investigated the relationship between the contrast of neuromelanin-sensitive MRI and three quantitative relaxation times using T_1_, T_2_, and T_2_* maps of the healthy human lower midbrain. The neuromelanin-sensitive MRI showed that the SNc region was a relatively high-signal region compared with the surrounding regions of the lower midbrain. The T_1_, T_2_, and T_2_* relaxation times were measured in detail using quantitative mapping. We found no significant correlations between the CR in the SNc and each relaxation time. Additionally, there were no significant correlations between the CRs in the other two regions (SCP and CP) and the relaxation times.

MTC is possible to improve contrast by decreasing the water proton SI where a tight magnetic coupling between water and macromolecules exists [31–33]. When using MTC preparation pulse at 3-Tesla or higher field, strong gradient field strength and wide bandwidth are required and often induce a relatively high specific absorption rate (SAR). Therefore, in this study, in order to protect human subjects from the extreme increase of the SAR by addition of MTC preparation pulse, a 2D-multi-slice T_1_WI-TSE sequence without MTC preparation pulse was utilized, which provides the equivalent magnetization transfer effect by refocusing pulse [23–25]. In this sequence, also called neuromelanin-sensitive MRI in this study, multiple refocusing pulses were applied resulting in off-resonance excitation in adjacent slices and hence magnetization transfer effects occurred [34]. This magnetization transfer effect is generally larger in white matter (such as SCP and CP) causing a greater signal reduction than in gray matter (such as SNc) in which the magnetization transfer effect is less [12]. Moreover, it is decreased in tissues containing paramagnetic substances such as iron [35, 36]. As a result, the signal from the surrounding tissues of the SNc was suppressed by the magnetization transfer effect.

Conventional MRI techniques have failed to depict neuromelanin-containing nuclei of the human midbrain [37]. The high-signal areas in the posteromedial portion of the CP contain neuromelanin and are visualized on the axial slice at the level of midbrain on neuromelanin-sensitive T_1_WI with MTC [38]. In our study, the ROIs were traced on a neuromelanin-sensitive MRI and on each quantitative relaxation map around four regions of the lower midbrain (Figs 1 and 2). The CR in the SNc using a T_1_-weighted 2D-TSE sequence, which was 0.056 ± 0.039, demonstrated that this region has a relatively high signal compared with the surrounding regions (Figs 1 and 3). For the calculation of the CRs on the neuromelanin-sensitive MRI, the MT was set as a reference ROI because it is located in the middle of the lower midbrain, where the magnetic field was considered to be the most homogeneous. There are several studies, which used various neuromelanin-sensitive sequences to estimate the CR in the SNc [9, 28, 30]. Sasaki et al. reported that the CR in the SNc was 20.3 ± 3.1% (0.203 ± 0.031), when using a T_1_-weighted 2D-fast SE sequence [9]. Another report by Watanabe et al. showed that a T_1_-weighted 3D-spoiled gradient-recalled acquisition in the steady state (spoiled GRASS) sequence with MTC pulse resulted in a CR of 9.60 ± 2.36% (0.096 ± 0.024) [28]. Langley et al. utilized a 2D-dual gradient-echo sequence with MTC preparation pulse and obtained the CR in the SNc, which was 0.16 ± 0.02 [30]. The value of the CR in the SNc seems to vary in different studies. The reasons for this include the different imaging sequences used, the presence or absence of the MTC preparation pulse, and the choice of the location of the reference ROI for calculating the CR in the SNc. Watanabe et al. and our study set the MT as a reference ROI, while Sasaki et al. set the decussation of the SCP. On the other hand, Langley et al. used the area outside the substantia nigra to define the reference ROI. Due to the above reasons, the CR in the SNc in this study was lower compared with their reports, however, our results showing that the SNc region has a relatively high signal compared with the surrounding regions were in accordance with these studies. Another previous report showed that high-resolution T_1_WI-TSE can capture the neuromelanin-generated signals from these nuclei, which show signal suppression of the surrounding brain tissue by T_1_ prolongation when using high-field MRI [39]. Hence, neuromelanin-sensitive MRI is considered useful for the direct visualization of the SNc as an intrinsic molecular probe in healthy volunteers.

Some neuromelanin-sensitive sequences utilizing MTC-based T_1_WI and T_2_-weighted image (T_2_WI) have been proposed for visualizing the SNc of the midbrain [8, 10, 11, 37, 40]. For example, neuromelanin-sensitive T_1_WI with MTC can distinctly visualize the SNc of the midbrain-level slice [38]. On the other hand, our neuromelanin-sensitive MRI used a 2D-multi-slice T_1_WI-TSE sequence without MTC preparation pulse. Regardless the low SAR due to no MTC pulse and the relatively short scan time (7 min 18 sec) compared with a previous report (12 min) [9], the resulting contrast was sufficient for visualizing the SNc as a hyperintense area. Visualizing the SNc by T_2_WI was not able to reliably differentiate the details of the midbrain [41], whereas proton density-weighted MRI in combination with short inversion time recovery imaging was able to accurately distinguish the SNc [42]. However, this previous study did not find a significant difference in the SNc volume in patients with PD [42]. Thus, the best MR signal contrasts to use in various neuromelanin-sensitive MRI methods are still being discussed [12].

Quantitative maps such as T_1_-LL enable the measurement of relaxation times for comparing MR images across subjects and scanners [43]. The T_1_-LL sequence is well known for being highly suitable for examining patients with acute myocardial infarction, because it allows for significantly shorter breath-hold time and provides a more accurate estimate of long T_1_ values at higher heart rates [44]. This sequence is also used for mapping various regions because of its rapid T_1_ mapping [45, 46]. Thus, in this study we applied the T_1_-LL sequence for the brain T_1_ mapping. In our study, the T_1_ relaxation times in the SNc were 856.4 ± 24.3 ms (Fig 4a), which is in agreement with a previous report [29]. According to this study, the T_1_ values in the left and right SNc were 877.35 ± 51.56 ms and 878.21 ± 42.22 ms, respectively. Although the T_1_ relaxation times in the SNc were higher than the relaxation times in the SCP and CP regions (Fig 4a, p < 0.001), the CR in the SNc showed a relatively high-signal region compared to these regions (Fig 3, p < 0.001). Dopaminergic neurons, which contain neuromelanin, are located primarily in the SNc [1] that appears as a hyperintense area on T_1_WI (Fig 1). This is because neuromelanin becomes paramagnetic when it combines with the iron deposited in the SNc resulting in T_1_-shortening effects [2, 7]. Even though a T_1_-shortening effect was expected, the T_1_ value in the SNc was observed to be higher than the surrounding regions (SCP and CP) on the T_1_ map in this study (Fig 4a, p < 0.001). This suggests that the high SI area on the neuromelanin-sensitive MRI does not reflect only the T_1_-shortening effect. It is possible that the magnetization transfer effect also contributes to the high SI area of the SNc. Moreover, there was no significant correlation between the CR in the SNc and the T_1_ relaxation times (Fig 5a, r = 0.21, p = 0.47). The CR in the other two regions also had no correlation with the T_1_ relaxation times (Fig 5b and 5c). Thus, it could be implied that the contrast in neuromelanin-sensitive imaging does not directly reflect the T_1_ contrast.

The T_2_ relaxation times usually had the same tendency as the T_2_* relaxation times [21, 22]. A previous study reported that the T_2_ value in the SNc was 81.9 ± 4.1 ms [47], which is similar to the T_2_ value we have found (Fig 4b, 76.1 ± 4.9 ms). A different study reported that the relaxation rate (R_2_*, R_2_* = 1/ T_2_*) in the SNc in healthy subjects was R_2_* = 32.8 ± 5.0 s^-1^, which is equivalent to T_2_* = 30.5 ± 4.6 ms [48]. This value is almost the same as our T_2_* value in the SNc (Fig 4c, 30.0 ± 3.5 ms). Both the T_2_ and T_2_* relaxation times in the SNc were lower than the relaxation times in the other three regions (Fig 4b and 4c). This implies the presence of iron deposition in the SNc. The T_2_* map reflects this deposition more than the T_2_ map. The 3-Tesla MRI scanner is sensitive to susceptibility effects, therefore it is believed that the iron deposition leads in a decrease in the T_2_* value due to the increased magnetic field heterogeneities caused by its paramagnetic effect [49]. As a result, the SNc appears as a hypointense area on the T_2_* map in our study and the reduced T_2_* values in the SNc suggest the existence of iron. However, there were no significant correlations between the CR and the T_2_ or T_2_* values (Fig 5d and 5g), showing that the neuromelanin-sensitive contrast is not directly affected by the amount of iron deposition. In this study, we used a high-field MRI resulting in susceptibility artifacts, which may have had a little influence on the T_2_* measurements [49]. Some promising techniques can correct the presence of geometrical and susceptibility artifacts of the human midbrain [50, 51]. Sophisticated sequences and techniques are needed for neuromelanin-sensitive MRI and for T_1_, T_2_, and T_2_* maps such as the 3D-TFE sequence [52], 3D-LL technique [53, 54], and rapid combined T_1_ and T_2_ mapping [55] in order to overcome these limitations and to improve the long acquisition time, reproducibility, and feasibility of neuromelanin-sensitive MRI and relaxation time mapping.

There were some limitations in this study, one of them being the fact that it was not possible to transfer the ROIs from the neuromelanin-sensitive image to the quantitative T_1_, T_2_, and T_2_* maps because the software used to analyze them was different. Furthermore, one person drew the ROIs manually to measure the SIs from the neuromelanin-sensitive MRI and the relaxation times from the maps. Because of this, the reproducibility of the ROI measurements was examined by taking more than one measurements for all ROIs in some of the subjects and it was confirmed that approximately the same values were obtained. One of the technical limitations of neuromelanin-sensitive MRI is the relatively low spatial resolution compared to the size of the SNc, particularly of the slice direction [9]. Also, the presence of signal non-uniformity arises from the heterogeneity of the magnetic field [56]. These technical problems may cause measurement errors in the quantitative analyses of signal alterations, particularly in small areas of the midbrain. There were other limitations in terms of image quality arising from not correcting both the B_0_ and B_1_ field heterogeneities. The midbrain location is affected by the magnetic field inhomogeneities and therefore inherent T_2_* and susceptibility effects are expected to be high in this location. It is believed that correcting the magnetic field heterogeneities would result in obtaining more accurate data. Recently, high 7-Tesla MRI with high-resolution T_2_*-weighted image, susceptibility-weighted image, and quantitative susceptibility mapping were used to visualize the SNc in healthy volunteers and patients with PD [57–59]. Quantitative assessments that combine these new methods and traditional quantitative methods have the potential to serve as a tool for visualizing and monitoring the alterations in the neuromelanin of the midbrain. These methods can be used to help with the long-term diagnoses of various disorders and aid in the development of treatments for patients with PD or AD.

To summarize, the SNc was visualized as a high SI area resulting in high CR compared with the surrounding regions such as the SCP and CP on the 2D-neuromelanin-sensitive MRI using a T_1_WI-TSE sequence. However, the T_1_-relaxation times in the SNc were longer, while the T_2_ and T_2_* values in the SNc were lower than them. There were no correlations between the semi-quantitative CRs on the neuromelanin-sensitive MRI including the CR in the SNc and the quantitative relaxation times. These results suggest that although the neuromelanin-sensitive MRI does not simply reflect the T_1_ contrast, both T_1_-shortening due to paramagnetic effects and magnetization transfer effects may contribute to the high SI area of the SNc on T_1_WI-TSE images. In conclusion, this study demonstrated the relationship between the CR of neuromelanin-sensitive MRI and the relaxation times of quantitative maps of the human lower midbrain. The quantitative evaluation can provide unbiased criteria for making comparisons between patients or healthy subjects, and between different MR scans, while the combination of quantitative and semi-quantitative evaluation has the potential to serve as a useful tool in future studies for further understanding the characteristics of neuromelanin-sensitive MRI.
